# The Diagnosis of Choriocarcinoma in Molar Pregnancies: A Revised Approach in Clinical Testing

**DOI:** 10.14740/jocmr2236w

**Published:** 2015-10-23

**Authors:** Lisa Duffy, Liangtao Zhang, Karen Sheath, Donald R. Love, Alice M. George

**Affiliations:** a Diagnostic Genetics, LabPLUS, Auckland City Hospital, Auckland, New Zealand

**Keywords:** Fluorescence *in situ* hybridization, Hydatidiform moles, Molar pregnancies, Ploidy

## Abstract

**Background:**

Hydatidiform moles occur in approximately 1 in 1,500 pregnancies; however, early miscarriages or spontaneous abortions may not be correctly identified as molar pregnancies due to poor differentiation of chorionic villi.

**Methods:**

The current clinical testing algorithm used for the detection of hydatidiform moles uses a combination of morphological analysis and p57 immunostaining followed by ploidy testing to establish a diagnosis of either a complete or partial molar pregnancy. We review here 198 referrals for fluorescence *in situ* hybridization (FISH) ploidy testing, where the initial diagnosis based on morphology is compared to the final diagnosis based on a combination of morphology, FISH and p57 immunohistochemical (IHC) staining.

**Results:**

Approximately 40% of cases were determined to be genetically abnormal, but only 28.8% of cases were diagnosed as molar pregnancies. The underestimation of complete molar pregnancies and those with androgenetic inheritance was also found to be likely using conventional diagnostic methods, as atypical p57 staining was observed in approximately 10% of cases.

**Conclusions:**

Our findings suggest that a revised approach to testing products of conception is necessary, with cases screened according to their clinical history in order to distinguish molar pregnancy referrals from hydropic pregnancies.

## Introduction

**Figure 1:**
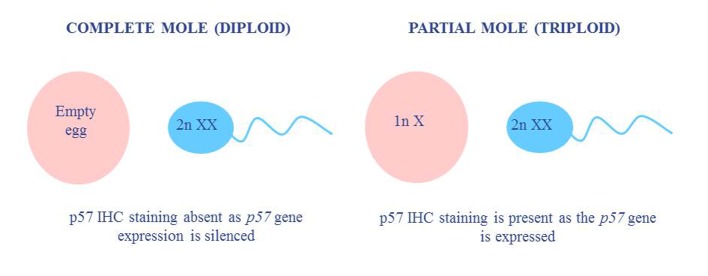
A comparison of the typical ploidy and p57 status in molar pregnancies.

Molar pregnancies occur in approximately 1/1,500 pregnancies in western populations [1, 2], and are generally detected by the second or third month of pregnancy, either by ultrasound or an elevated human chorionic gonadotropin (hCg) level [1, 3]. In such pregnancies, an abnormal placental mass develops as a result of a diandric genome (a diploid paternal complement) following an unviable fertilization event [4]. In the case of a complete mole, an empty egg is fertilized by either a single sperm in which the genome has undergone replication, or two individual sperm, causing the absence of a maternal genomic complement (Fig. 1, Table 1) [5]. Complete moles are characterized by grossly swollen villi, giving the placenta an appearance of a bunch of grapes, which can make it hard to differentiate histologically from a hydropic abortus. Hydropic degeneration of villi may also occur following spontaneous abortion of a biparental pregnancy due to abnormal development of villus vasculature [6].

**Table 1:** A Comparison of the Characteristics of Partial and Complete Moles (Derived From [5])

Characteristic	Partial mole	Complete mole
Development	Some fetal development but malformed	No fetal development (cord, membrane present)
Villi	Some enlarged; two populations of villi Blood vessels and fetal red blood cells present Scalloped outlines with pseudoinclusions and invaginations Focal trophoblast proliferation Minimal trophoblast atypia	All enlarged Blood vessels absent Round to ovoid Prominent cisterns Circumferential trophoblastic proliferation Trophoblast atypia present
Origin	Both maternal and paternal	Paternal only
Genetics	Triploid	Diploid (or tetraploid)
p57 IHC stain	Positive	Negative

In partial moles, a normal egg is fertilized by either a single sperm in which the genome has undergone replication, or two individual sperm, leading to a triploid genomic complement (Fig. 1, Table 1). The presence of a maternal complement means that only some of the villi show swelling, leading to two morphologically different populations within the same sample. The villi also show characteristic features such as scalloping, cistern formation and trophoblastic proliferation, and are thus easier to detect than complete moles. These characteristic features are less identifiable in early pregnancy loss, however, as villi earlier than 12 weeks gestation may not show typical features of a molar pregnancy and can therefore be hard to diagnose [5]. The best results are achieved when p57 immunohistochemical (IHC) staining and fluorescence *in situ* hybridization (FISH) ploidy testing are performed in conjunction with morphological analysis (Table 1).

The p57^KIP2^ IHC stain is a nuclear stain that is used to detect diandry. The *p57* gene is maternally expressed and paternally methylated [7]. In a normal pregnancy with biparental inheritance, the p57 stain would be positive due to the presence of a maternal genetic complement, whereas a diandric complete molar pregnancy would show absence of staining due to the epigenetic silencing of the *p57* gene. p57 staining is usually performed in conjunction with FISH ploidy testing in order to detect triploid partial molar pregnancies. Such pregnancies would still show positive p57 staining due to the presence of a maternal genomic complement, so the assessment of ploidy is required to avoid being scored as a diploid pregnancy.

The identification and classification of molar pregnancies is desirable due to a 15% risk of choriocarcinoma development in the case of a complete mole, compared to a 0.5% risk for a partial mole [4]. A choriocarcinoma occurs when cells from the abnormal placental mass become cancerous and metastasize to other parts of the body such as the uterus, lungs or brain [1]. The *p57* gene is a candidate tumor suppressor gene, therefore silencing of the gene in a molar pregnancy leads to either functional nullisomy or monosomy, and the potential expression of other recessive oncogenes. Without the detection and successful treatment of a complete mole, subsequent pregnancy attempts can cause symptoms to worsen, leading to TB-like symptoms if the lungs are infiltrated, or headaches, dizziness and seizures if the brain is infiltrated [1]. Treatment is generally successful in greater than 90% of cases, so early detection is desirable [1].

In this study, we analyzed both the clinical referral reasons and the results of combined morphological analysis, FISH and p57 staining in order to estimate the frequency and detection rate of molar pregnancies, and to assess whether such an approach provides an effective detection strategy for hydatidiform moles and choriocarcinomas.

## Methods

One hundred and ninety-eight molar pregnancy FISH testing referrals were reviewed, with pathology reports evaluated before and after FISH testing to assess the accuracy of diagnosis by morphology. A second analysis was also performed on 104 cases in which the p57 IHC staining result was available, thereby allowing an estimate of the level of concordance between FISH results and *p57* gene expression.

Two FISH slides were prepared for each case by first aging the slides for 20 min in a 60 °C oven, then placing them in xylene for 15 min to remove the wax. They were next placed in a 100%, 80%, and 70% ethanol series for 2 min each, ending with 2 min in deionized water at room temperature. Slides were drained briefly, 30 - 60 μL of 0.2 M HCl solution was pipetted on to each of the slides, and then they were covered with parafilm and incubated in a humidity chamber at 37 °C for 20 min. Following this, slides were rinsed in a coplin jar of deionized water and drained, then 30 - 60 μL of heat pretreatment solution (SPoT-Light Tissue Pretreatment Kit) was pipetted onto the slides. They were then covered with a glass coverslip, and sealed with rubber cement. Slides were heated on the thermal cycler for 30 min at 95 °C and then the coverslip was gently removed and the slides immersed in deionized water. After draining briefly, 15 - 30 μL of enzyme reagent (SPoT-Light Tissue Pretreatment Kit) was added to the slides, they were covered with a square of parafilm, and incubated for 30 min in a humidified chamber at 37 °C. The parafilm was then removed, the slides washed briefly in a coplin jar of deionized water at room temperature, and finally dehydrated for 2 - 3 min each in each of 70%, 80% and 100% ethanol solution and air dried at room temperature.

The pretreated paraffin slides were matched against a corresponding marked H&E slide (Shandon Rapid-Chrome^TM^ Frozen Section Staining kit), and the area for testing transferred to the pretreated slides using a marker pen initially, followed by a diamond-tipped engraver. The XY18 and 13,21 Vysis aneuscreen probe sets (Abbott Molecular) were then applied to the hybridization site marked on each slide, a glass coverslip applied, and the area was sealed with rubber cement. The slides were denatured in the thermal cycler slide for 20 min at 85 °C, and then placed in a humidified box in the incubator at 37 °C for 12 - 16 h. A post hybridization wash was performed by gently removing the rubber cement, and then washing slides in a coplin jar of 0.4 × SSC/0.03% NP40 solution at 72 °C for 2 min, before being transferred to 2 × SSC/0.01% NP40 at room temperature for 30 s. Slides were then drained, Vectashield with DAPI and a coverslip applied, and finally visualized under a fluorescence microscope. Slides were analyzed by two observers, each analyzing a minimum of four different areas within the targeted area.

## Results

Prior to FISH testing, pathology referrals fell into three main groups (Table 2): those querying a molar pregnancy (70/198), those wanting to exclude a molar pregnancy (39/198), and those that queried a hydropic pregnancy (89/198). After FISH testing, however, 57/198 (28.8%) were diagnosed as molar pregnancies, with the majority (122/198 or 61.6%) classed as hydropic (Table 2). This result shows that the diagnosis of molar pregnancies by morphology is less accurate than the diagnosis of hydropic pregnancies (Table 2).

**Table 2:** Referral Reasons for Ploidy Testing Based on Morphology Compared to the Final Diagnosis Using FISH

	Initial referral reason			
	Molar pregnancy referral (n = 70)	Exclude molar pregnancy referral (n = 39)	Hydropic/Other referral (n = 89)	Total (n = 198)
Final diagnosis				
Molar pregnancy	40	5	12	57
Hydropic pregnancy	23	30	69	122
Other	7	4	8	19
Percentage correctly diagnosed (%)	57.1%	87.2%	86.5%	

In total, 82/198 (41.4%) cases were determined to be genetically abnormal, either as a molar pregnancy or an aneuploid conceptus. Among the 82 abnormal cases, 4.5% (9/198) were classified as complete moles and 24.2% (48/198) were classed as partial moles, with the remaining 25/198 (12.6%) cases reported as aneuploid. The aneuploidy cases were trisomy 13 (six cases), trisomy 18 (seven cases), trisomy 21 (four cases), monosomy 21 (two cases), monosomy X (five cases) and a double trisomy for chromosomes 13 and 18.

The ploidy results were generally concordant with the p57 IHC staining, although atypical p57 staining was observed in 10/104 (9.6%) cases overall (Table 3). When these atypical cases were examined, 8/10 showed an absence of p57 IHC staining (Table 3), indicating androgenetic inheritance. Despite this, the final diagnosis was made based on the ploidy level of the sample, rather than inheritance pattern in 9/10 cases (Table 3), so the percentage of cases with androgenetic inheritance is likely to be underestimated. The risk of underestimation due to discordance between the IHC and ploidy increased when only the cases diagnosed as molar pregnancies were assessed. In total, 27/104 (26.0%) cases were diagnosed as molar pregnancies, but out of 26 which showed definitive p57 staining, 6/27 (22.2%) of these cases showed discordance between the IHC and FISH results (Table 4). Closer examination showed that three of the atypical cases had atypical staining in the cytotrophoblast (Table 4), suggesting the presence of different cell lines.

**Table 3:** A Summary of the Cases Showing Atypical p57 IHC Staining Patterns

Category	P57 stain	Sex	Other abnormality?	Maternal genome status and possible causal mechanism
Complete mole	Positive	XX	No	Present - retained maternal chromosome 11/trisomic rescue
Partial mole	Negative	XXY	Triploid	Absent - dispermy or mutation in maternal p57 allele
	Negative	XXX	Triploid	Absent - dispermy or mutation in maternal p57 allele
	Positive	XX	No	Present - biparental mole
	Negative	XXY	Triploid	Absent - dispermy or mutation in maternal p57 allele
	Negative	XXY	Triploid	Absent - dispermy or mutation in maternal p57 allele
Hydropic	Negative	XY	T18	Absent - dispermy, biparental mole or mutation in maternal p57 allele
	Negative	XX	T21	Absent - dispermy, biparental mole or mutation in maternal p57 allele
	Negative	XY	No	Absent - dispermy, biparental mole or mutation in maternal p57 allele
Other	Negative	XX	No	Absent - dispermy, biparental mole or mutation in maternal p57 allele

**Table 4:** A Comparison of the Sex Complement and p57 IHC Staining Results in Molar Pregnancies

Category	Sex complement	Number of cases	p57 stain
Complete mole (p57-)	XX	8	One case atypical (p57+): focally positive staining in the cytotrophoblast
	XY	0	
Partial mole (p57+)	XXY	14	Three cases atypical: two were p57 negative, and one showed absent staining in the basal cytotrophoblast
	XYY	1	Suboptimal
	XXX	3	One case atypical: p57 negative
	XX	1	One case: p57 positive
Total		27	7

## Discussion

The results reported here highlight a need for the clear differentiation of genetic disorders that result in fetal demise, and those that are detrimental to the mother. Fetal demise may occur for a number of reasons, but molar pregnancies put the health of the mother at risk, and therefore need to be accurately diagnosed and treated. Approximately 10-15% of molar pregnancies are invasive, causing hemorrhaging and other complications, with 2-3% developing into a choriocarcinoma [1, 4]. Diagnosis can be difficult, particularly in early pregnancy loss, when many of the characteristic features of molar pregnancies are ill-defined in the villi due to poor tissue differentiation [5].

The analysis of the initial referral reasons for FISH ploidy testing showed that the majority of referrals (60.6%) used FISH testing as an exclusion tool, rather than for a definitive diagnosis of the disease (Table 2). As 44.9% of samples in this study were querying hydropic villi rather than molar pregnancies at the outset, and 71.2% of pregnancies were later found to be hydropic or non-molar, an alternative method of genetic testing may have been more appropriate at the outset.

**Figure 2:**
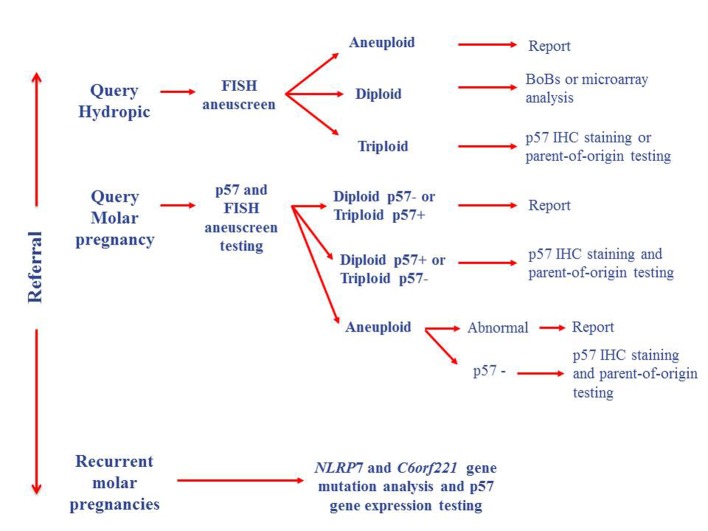
Proposed testing algorithm for products of conception.

BACs-on-Beads (BoBs) and microarray technologies [8-10] have been successfully used in a number of centers for the testing of products of conception, thus increasing the detection rate of genetic abnormalities. These techniques could be combined with genotyping [11] and mutational analysis in apparently normal cases to test for inheritance disorders (Fig. 2). Hydatidiform moles are considered to be a disorder of genomic imprinting [12], therefore genotyping on its own is insufficient for the diagnosis of molar pregnancies [12], and may be of limited use if DNA quality is poor or maternal contamination is greater than 20% [11].

Current testing methods are likely to underestimate the frequency of both complete moles and pregnancies with diandric inheritance. In this study, complete moles had the lowest detection rate, which could either reflect the rarity of such an event, or inadequacy of the p57 stain as a definitive test for androgenetic inheritance. Approximately 10% of referrals in this study showed atypical p57 staining patterns (Table 3), and the majority showed loss of expression, suggesting either the complete absence of a maternal genome in the fetus, or that loss of the p57 function had occurred via an alternative mechanism. The absence of p57 staining occurred in four cases diagnosed as hydropic abortuses, which may in fact have been complete moles due to either uniparental disomy (UPD) for chromosome 11, or a mutation in the maternal copy of the *p57* gene (Table 3). The absence of staining was also observed in four molar pregnancy cases (Table 3), all of which were triploid partial moles (Table 3), suggesting loss of the maternal genomic complement from either a p57 mutation or dispermy. The latter would be unsurprising, as Zaragoza et al [13] reported that approximately two-thirds of triploids are of paternal origin due to dispermy, with a minority (8.3%) due to fertilization by a diploid sperm [4, 14].

Without molecular genetic studies, it is not possible to determine the absence of p57 staining. This absence may be due to diandric inheritance, UPD for chromosome 11, or to biparental inheritance with a mutation in the maternal *p57* (*CDKN1C*) gene. The *p57* gene is located in the 11p15 region, and mutations of the maternal 11p15.5 *p57* gene (OMIM 600856) are associated with Silver-Russell syndrome (SRS). Mutations associated with SRS are most frequently caused by loss of methylation in the differentially methylated region 1 (DMR1) at 11p15 (50%), and mutations or aberrant methylation at the maternal chromosome 11p15 locus are linked to growth retardation, hemihypoplasia, and an increased association with cancer, macrosomia and midline abdominal wall defects, and several other childhood tumors [4], such as Wilms’ tumor (OMIM #194071), adrenocortical carcinoma (OMIM #202300), and rhabdomyosarcoma (OMIM #268210). This may explain the increased risk of choriocarcinoma in complete molar pregnancies compared to those with digenic inheritance or paternal UPD of chromosome 11, which causes the opposite overgrowth disorder, Beckwith-Wiedemann syndrome (BWS).

Establishing the parent-of-origin of gene expression imbalance is therefore of importance in the diagnosis of molar pregnancies in order to discern the risk of choriocarcinoma development. A complete mole with p57 positivity was observed in this study (Table 3), suggesting either retention of the maternal chromosome 11 [15], or a biparental complete mole. Biparental complete moles are generally the result of abnormal methylation of maternal alleles [16] and usually show complete lack of p57 staining [12], although maternal homozygous or compound heterozygous mutations in the *NLRP7* and *C6orf221 (KHDC3L)* genes have also been implicated when fetuses are shown to be diploid and biparental in origin [17-19]. Both the 6q13 *C6orf221* (OMIM #614293) and 19q13.42 *NLRP7* (OMIM #609661) genes are expressed in oocytes, and are thought to be involved in setting and/or maintaining the maternal imprint [19], with mutations in the latter thought to be linked to early cleavage errors and subsequent reduced maternal immune response that allows abnormal cells to persist [18]. Maternal imprinting defects or non-synonymous or stop mutations in the *NLPR7* gene [2, 12, 18] have also been suggested as the cause of recurrent molar pregnancies. Therefore testing methods that are able to establish parental inheritance and also distinguish between gene mutations or epimutations are desirable, as these would provide an estimate of both the risk of choriocarcinoma development and the recurrence risk of such pregnancies. This would be particularly relevant to fertility or recurrent miscarriage referrals, as diandric triploids are the most frequent chromosome abnormality in infertile males with oligo-, crypto- and azoospermia [14].

The clinical history of the patient should therefore be used to guide testing options (Fig. 2), as patients with a history of molar pregnancies and young recurrent miscarriage couples should be tested at the outset for mutations in the *NLRP7* and *C6orf221* genes, and *p57* gene expression levels, in order to establish whether mutations or epimutations in these genes are occurring. This would provide information about recurrence risk for future pregnancies, and the risk of choriocarcinoma due to imprinting defects. Cases with no prior clinical history and a morphological diagnosis of a molar pregnancy would be better suited to initial screening by p57 staining and FISH ploidy testing, with atypical cases followed up by parent-of-origin testing to establish whether uniparental disomy has occurred, and also methylation analysis of the *p57* gene to assess the risk of choriocarcinoma development. BoBs or microarray testing methods are more appropriate for the detection of aneuploidy or sequence variants in all other cases, combined with p57 IHC staining to provide an indicator of imprinting defects that could be followed up by other methods.

Present testing methods such as ploidy and p57 staining may therefore both underestimate the incidence of complete molar pregnancies and also the complexity of androgenetic inheritance. These methods rely on either the absence of expression of the maternally expressed *p57* gene, or the inference of *p57* gene expression from the combined assessment of the sex complement of the fetus and the villus morphology. Although they can provide an effective initial screen for molar pregnancies, they should not be used for general early pregnancy losses which are more likely to be due to aneuploid errors. Cases showing discordant results should be followed up by combined parent-of-origin and methylation studies. A revised approach to testing would provide more accurate diagnosis of molar pregnancies, and also allow an estimate of the recurrence risk for couples.
